# Processing Effects on the Martensitic Transformation and Related Properties in the Ni_55_Fe_18_Nd_2_Ga_25_ Ferromagnetic Shape Memory Alloy

**DOI:** 10.3390/nano12203667

**Published:** 2022-10-19

**Authors:** Mihaela Sofronie, Bogdan Popescu, Monica Enculescu, Mugurel Tolea, Felicia Tolea

**Affiliations:** National Institute of Materials Physics, Atomistilor 405A, 077125 Magurele, Romania

**Keywords:** Heusler FSMA, nanostructural processing effects, melt spinning, martensitic transformation, magnetocaloric effect, magnetoresistive effect

## Abstract

The influence of processing on the martensitic transformation and related magnetic properties of the Ni_55_Fe_18_Nd_2_Ga_25_ ferromagnetic shape memory alloy, as bulk and ribbons prepared by the melt spinning method and subjected to different thermal treatments, is investigated. Structural, calorimetric, and magnetic characterizations are performed. Thermal treatment at 1173 K induces a decrease in both the Curie and the martensitic transformation temperatures, while a treatment at 673 K produces the structural ordering of the ribbons, hence an increase in T_C_. A maximum value of the magnetic entropy variation of −5.41 J/kgK was recorded at 310 K for the as quenched ribbons. The evaluation of the magnetoresistive effect shows a remarkable value of −13.5% at 275 K on the bulk sample, which is much higher than in the ribbons.

## 1. Introduction

The Ferromagnetic Shape-Memory Alloys (FSMA) are intensively investigated due to their diversified application potential in various fields such as: biomedical [1,2]; civil structures [3]; miniature devices and robotics [4,5]; and other applications [6,7,8]. At temperatures lower than the magnetic ordering temperatures they undergo the so-called martensitic transformation (MT); this is a thermoelastic, reversible, and diffusionless transformation, which occurs between a high temperature crystalline phase of high symmetry (austenite) and a low temperature one of low symmetry (martensite) [9].

An important class of FSMA are the Heusler alloys which incorporate a huge number of magnetic members exhibiting diverse magnetic phenomena such as itinerant and localized magnetism, antiferromagnetism, helimagnetism, Pauli paramagnetism, or heavy-fermionic behavior [10,11]. Ni_2_MnGa is a representative and the most investigated member of this class of materials, which shows a huge magnetic-field-induced strain (MFIS) below the austenite–martensite transformation, of the order of several percent, as evidenced by Ullakko et al. [12]. However, the compound’s brittleness makes it difficult to be used in applications, and for this reason the search for new Heusler alloys that undergo an MT was extended comprising Co-Ni-Ga [13,14,15] and Ni-Mn-(Al, In, Sn) [16,17,18,19,20,21]. Ni-Fe-Ga alloys [22,23,24,25,26] have emerged as good candidates for replacing the brittle Ni-Mn-Ga in applications [27]. They show enhanced mechanical durability during the reversible austenite–martensite transformation and exceptional magnetoelastic properties promoted by their structural twinning. The improved ductility of the off-stoichiometric Ni-Fe-Ga alloys is caused by the existence of secondary phases [28], which may be controlled to some extent by suitable preparation techniques, e.g., melt spinning, where the obtained ribbons preserve a relatively good sample malleability. In addition, it is assumed that the thin melt-spun ribbons assure a more efficient heat transfer, which is desirable in magnetocaloric applications. In particular, the sample’s malleability is caused by a face-centered cubic gamma phase, which forms during the heat treatments necessary for sample structural ordering. Its presence, however, might alter the intrinsic properties of the alloy and, therefore, single phase alloys obtained by ultrafast cooling are desirable. Another approach for improving the magnetic and mechanical properties of Heusler type FSMA is by substituting with Rare Earth (RE) elements [29,30,31,32]. Thus, in Ni-Mn-Ga alloys various substitutions have been tested in order to improve their magnetic anisotropy and ductility. Research shows that the low solubility of the RE in the alloy matrix (below 0.1 at%) produce no major changes in the MT characteristics; instead new phases precipitate at the grain boundaries that positively impact the cohesion between grains. For instance, it was observed that the addition of RE (Nd, Sm, Tb) to the Ni-Mn-Ga [33] shape memory alloy significantly improves the compressive ductility of the alloy. The results obtained in the latter case also suggest the suitability of RE substitution approach in Ni-Fe-Ga based alloys, which might corroborate previous investigations on the influence of different quenching rates on the structural ordering of Ni_2_FeGa melt spun ribbons [34]. In addition, deviating from the exact composition (Ni_50_Fe_25_Ga_25_) has a tuning effect (higher/lower Curie or structural transition temperatures). In our case, one purpose was to search for concomitant magnetic and structural transitions, which has been achieved for the as-quenched ribbons, as shall be described.

The magnetocaloric effect (MCE) is defined as the heating or cooling of a magnetic material, isolated adiabatically, when a magnetic field is applied and is due to the correlation that exists between the magnetization state of a system and its entropy. In the context of global warming, materials with MCE around room temperature are an environmentally friendly alternative to conventional refrigeration. The giant magnetocaloric effect observed on Gd_5_Ge_4_ compounds [35] was explained based on the coincidence between the magnetic ordering temperature and the crystallographic transformation temperature. From this point of view, Heusler-type shape memory ferromagnetic alloys are interesting. Both the Curie temperature and the martensitic one can be modified by slight changes in the composition and using proper thermal treatments. Thus, the structural transformation can overlap with the magnetic one so that the alloy has a magnetostructural transformation. In the present paper, we evaluate the magnetic entropy changes (ΔS_m_) in the Ni_55_Fe_18_Nd_2_Ga_25_ alloy with concomitant or sequential structural and magnetic phase transition prepared as bulk and as ribbons in an as quenched state or subjected to thermal treatments.

Generally, the MT temperatures are correlated with a large number of parameters that might influence the transformation, such as the processing route [36], chemical and atomic order, or internal stress [37]. For this reason, various preparation routes have been tested in order to improve the magnetic or mechanical properties of FSMA alloys [22,38,39], together with complementary suitable thermal treatments to tailor the working temperature range and performance of FSMA. Thus, in this study, the properties of Ni-Fe-Ga with Nd substitutions synthesized by arc melting and melt spinning and subjected to several thermal treatments are investigated. By analyzing the bulk and, respectively, the ribbons’ properties, we expect to infer the role of preparation route, interfaces, and the microstructure, in what regards the crystalline phases present, the martensitic transformation (MT), and the magnetocaloric (MCE) and magnetoresistive (MR) effects.

## 2. Materials and Methods

The constituting high purity elements were weighed according to the nominal composition Ni_55_Nd_2_Fe_18_Ga_25_, and melted in an electric arc furnace under argon protective atmosphere. The samples were melted 5 times flipping the ingots in order to ensure homogeneity. The resulting ingots were cut using a low speed diamond saw and a small piece was thermally treated in high vacuum for 25 h at 1223 K, followed by quenching in iced water. The remaining ingot was subjected to melt spinning technique and inductively melted in a quartz tube under argon atmosphere, and ejected under a 40 kPa of Ar overpressure, through a nozzle of 0.5 mm in diameter, on a water-cooled copper wheel rotating at a surface velocity speed of 20 m/s. Rapidly quenched ribbons of about 15–16 µm thickness and 2–3 mm width were obtained and denoted AQ (“as quenched”). The AQ ribbons were thermally annealed in evacuated quartz ampoules followed by a direct quenching of the sample in ice water. The samples were annealed for 2 h at 673 K, and for 2 min at 1173 K and denoted T400 and T900, respectively.

The phase transitions and associated transformation temperatures of the samples were determined using a differential scanning calorimeter (DSC) model 204 F1 Phoenix (Netzsch), in the temperature range 200–400 K with a 20 K/min scanning rate. The structural investigations were carried out by room temperature (RT = 290 K) X-ray diffraction (XRD) using a Bruker D8 Advance X-ray diffractometer (Hamburg, Germany) in Bragg–Brentano geometry (Cu Kα = 1.5406 Å radiation) and lithium fluoride (LiF) monochromator. The phase composition of samples was evaluated using Bruker AXS DIFFRAC. EVA software (Bruker AXS, Karlsruhe, Germany, 2000), while the LeBail refinement method of the XRD-data was performed using the FullProf Suite software. The morphology and composition of the samples were investigated by Scanning Electron Microscopy (SEM) and Energy Dispersive X-ray Spectroscopy (EDS), respectively, employing a Zeiss Evo 50 XVP microscope. The SEM images were taken with a secondary electrons detector without chemical or ion polishing of the surfaces. The acceleration voltage of SEM is EHT = 20 kV. Magnetic measurements were performed by a Quantum Design superconducting quantum interference device (SQUID) (San Diego, CA, USA) in the Reciprocal Space Option (RSO) mode. The magnetic field was applied along the ribbon length in order to minimize the demagnetization effect. The dependences of the electrical resistivity versus temperature were performed in standard four-probe method using a Quantum Design Physical Property Measurement System (QD-PPMS), with the current applied along the longitudinal direction of the ribbons and the magnetic field perpendicular to the ribbons.

## 3. Results and Discussions

### 3.1. DSC

**Figure 1:**
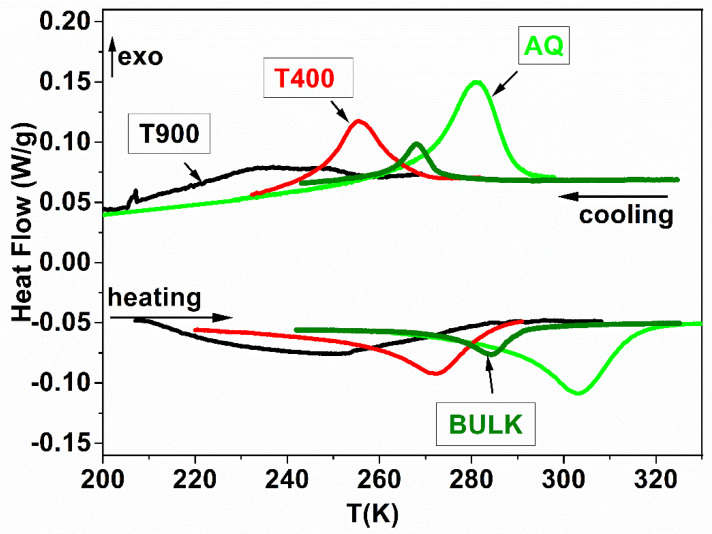
The DSC signals for Bulk and AQ, T400 and T900 ribbons.

Reversible thermoelastic transformations are revealed by the DSC signal for all investigated samples (Figure 1). The temperatures that define the martensitic transformation are shown in Table 1: start austenite (As); final austenite (Af); start martensite (Ms); final martensite (Mf); and thermodynamic equilibrium temperature T_0_. The thermodynamic equilibrium temperature is the one at which the Gibbs energies of martensite and austenite are equal and can be estimated by the relation T_0_ = (Ms + Af)/2 [28]. In Figure 1, it is obvious that the martensitic transformation temperatures decrease as the heat treatment temperature increases. AQ ribbons exhibit not only higher MT temperatures but also the highest heat of transformation. Thus, the average value between the direct and reverse transformation heat of the AQ ribbons is approximately double (Q = 2.1 J/g) compared to the bulk alloys which have Q = 1.07 J/g [40]. This behavior is the effect of the internal stress retained in the ribbons during rapid cooling which acts as an additional driving force for MT.

### 3.2. XRD

**Figure 2:**
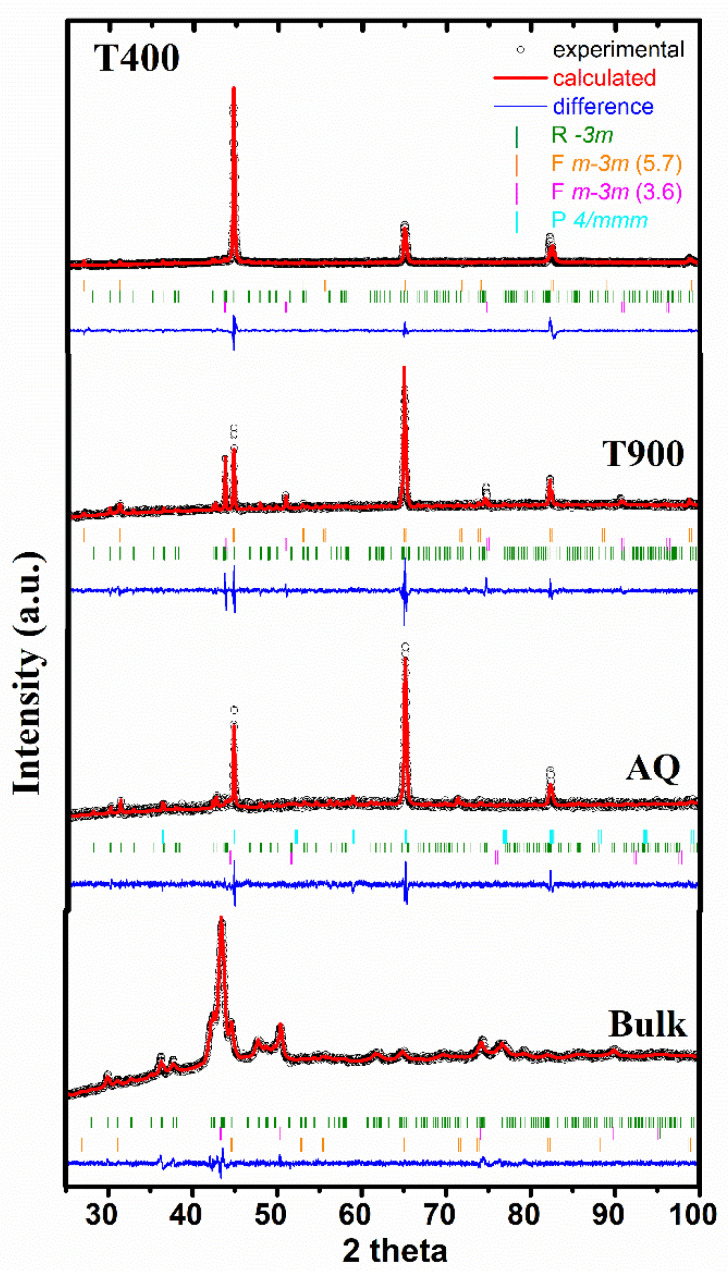
XRD spectra recorded at RT.

Le Bail fits to the XRD-data collected at room temperature (RT) on bulk, AQ, T400 and T900 samples are shown in Figure 2. The crystalline structure of all samples is complex, and is composed of three phases that grow under the influence of the processing route and the applied thermal treatment. The phase composition of bulk samples (Figure 2) consists of two-face-centered cubic crystal phases (Fm-3m) with different lattice parameters (see Table 2) and one rhombohedral (R-3m). The same phase composition is also detected in the thermally treated ribbons having different mean crystallite sizes. Notably, the austenite cubic phase with a ~3.6 Å and the rhombohedral Nd_2_Fe_17_ type crystalline phase are detected in all samples. In the AQ ribbons, due to the fast cooling rate during synthesis, the growth of γ phase is inhibited but the tetragonal martensitic P4/mmm phase is “frozen”. Applying a thermal treatment to ribbons produces an atom reordering and the segregation of the cubic γ phase (Fm-3m, with a ~5.7 Å) corroborating the DSC results, which suggest that thermal treatments stabilize the austenite phase and lower the martensitic transformation temperatures [41,42].

The mean crystallite size of each crystalline phase (see Table 2) was calculated according to the Scherrer relation D_p_ = K *λ*/*β* cos*θ*, where K is the Scherrer constant (here 0.94), *λ* the wavelength, *β* is the half width of a reflection peak, and *θ* the diffraction angle. In the AQ ribbons, the ultra-fast cooling prevents the crystallite growth and consequently the samples are nanostructured with the mean crystallite size of only several tens of nm. Importantly, for the rhombohedral Nd_2_Fe_17_ phase, the crystallite size exceeds 100 nm in all samples, but as suggested by the SEM investigations, the phase abundance is reduced and might not exert an important influence on sample properties. Besides atomic reordering, the thermal treatments promote the precipitation of the γ phase on the expense of the other phases, which deprives the austenitic matrix of Fe and Ni atoms, and further reduces the valence electron concentration and the MT temperature [42]. However, the increase in the average crystallite size of the γ phase with the temperature of the TT and its concomitant diminishing for the other two phases, might also be a consequence of the austenite matrix depletion in Fe and Ni as more of these atoms will participate to the γ phase forming.

### 3.3. SEM

**Figure 3:**
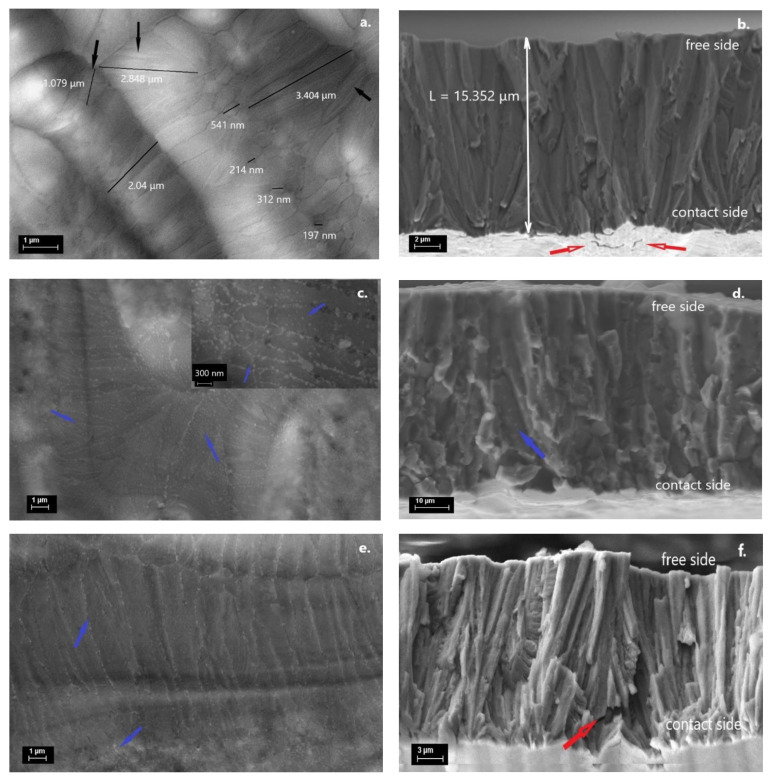
SEM images for AQ (**a**), T2-900 (**c**) (Inset with enlarged image), T1-400, (**e**) samples, with their cross-section images (**b**,**d**,**f**). The twin microstructure is indicated with black arrows (**a**), the small cracks are shown with red arrows (**b**,**f**) and the precipitates with blue ones (**c**,**d**,**e**).

The surface and cross-section morphology at room temperature of all samples were investigated by scanning electron microscopy (SEM) with secondary electrons detector without chemical or ion polishing of the surfaces (Figure 3). The surface morphology of the AQ ribbons is typical for samples produced by melt spinning, which involves very fast cooling rates, consisting of dendritic grains with length of micron size (0.2–3.5 μm) and a visible twin microstructure (shown with black arrows), and twin thickness of several tens of nanometers. The cross-section image (Figure 3b) shows small cracks on the contact side surface (indicated by red arrows) and the existence of columnar grains, which span the entire thickness of the ribbon, a signature of temperature gradient typical for the melt-spinning technique, which induces a fast nucleation and growth process of grains along the cross-section during the rapid cooling of the melt [43]. Although the XRD suggests the existence of a Nd_2_Fe_17_ type phase, the SEM images show no clear signs of precipitates. This might be explained by the reduced amount of Nd_2_Fe_17_ phase in the ribbons and that the phase forms mostly at the grain boundaries.

As expected, on the surface of the heat-treated ribbons at high temperatures (1173 K), the γ phase precipitates inside and at the grain boundaries (indicated by the blue arrows in Figure 3c). The inset of Figure 3c shows precipitates of nanometric dimensions (~60 nm) increasing in size along the dendritic grains (~100 nm). Nevertheless, the precipitates are causing the crack’s disappearance because the gamma phase is responsible for the improved alloy ductility [28]. Similarly, the heat treatment at a lower temperature (673 K) yield nanometric precipitates of the developing γ phase at the grain boundaries (Figure 3e). The cross-section investigation shows a well-defined columnar structure with cracks at the contact side of the ribbons (Figure 3f). EDX analysis indicates that the chemical compositions are the nominal ones (within the limits of the method accuracy) for all samples.

### 3.4. Magnetic Properties

**Figure 4:**
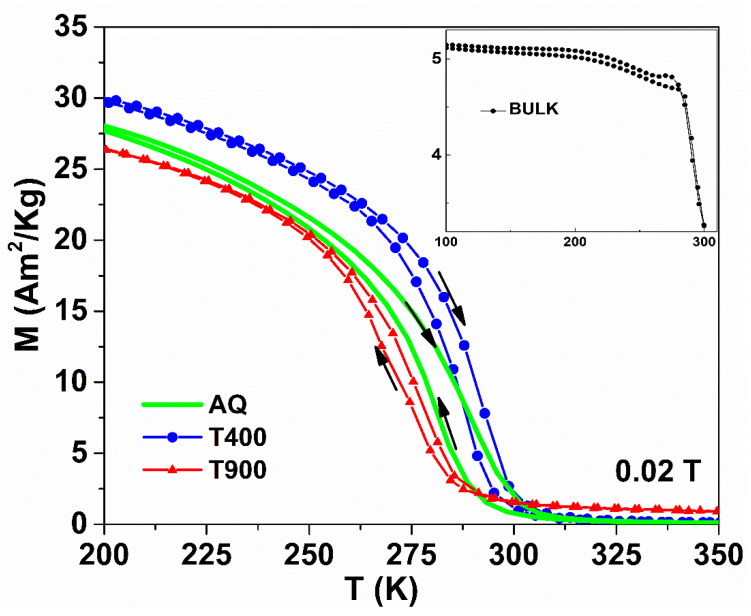
Temperature dependence of magnetization for AQ, T400 and T900 ribbons and for Bulk in inset.

The hysteresis of the thermomagnetic curves recorded on cooling and heating, at low magnetic fields (0.02 T), describe the martensitic transformation of all studied samples (see Figure 4). Thermal treatments produce specific changes to the characteristic temperatures of the martensitic transformation. The 2 h treatment at 673 K induces the structural ordering, and the stress relaxation caused by the ultra-rapid cooling of the ribbons, which determines the increase in the MT temperatures, and of the Curie temperature of the T400 sample. As highlighted by the XRD patterns and SEM images, the heat treatment performed at 1173 K promotes the growth of the secondary γ phase. The γ phase has an essential role in lowering the MT temperature [42], and its presence is also indicated by the higher Curie temperature (T_C_ > 350 K) in the T900 ribbons (red curve from the thermomagnetic measurements). The fact that T_C_ and MT of the AQ and Bulk samples are in the same temperature range may be of great interest for applications. A first-order transition around room temperature associated with the magnetic transition (magnetostructural transformation) is of interest for the use of the associated magnetic entropy change in practical applications.

### 3.5. Evaluation of Magnetocaloric Effect

**Figure 5:**
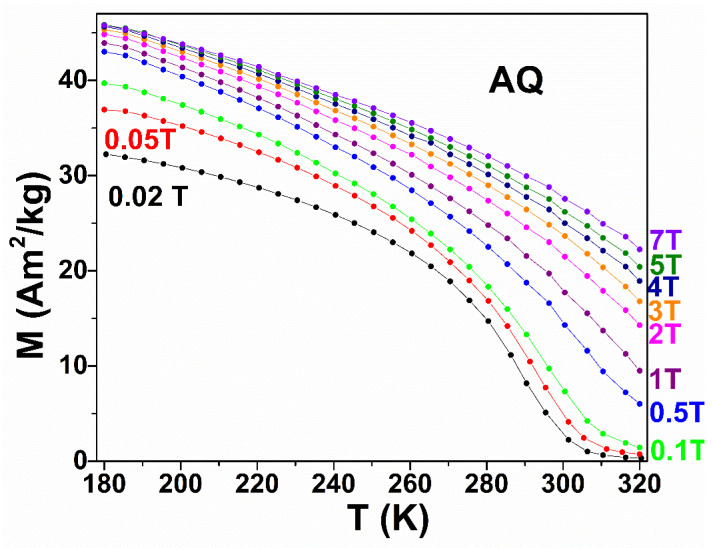
Temperature dependence of magnetization at different applied magnetic fields for AQ ribbons.

Here, a commonly indirect measurement technique to assess MCE is used. Magnetization was measured as a function of increasing temperature for applied magnetic field values between 0.02 T and 7 T; depending on the measurement accuracy an error of only 3–10% for the magnetic entropy change may be achieved. In Figure 5, such an example of magnetization measured on the AQ ribbons is shown. From the temperature dependence of the magnetization in the applied constant magnetic field, the magnetic entropy variation is calculated using the Maxwell equation for discrete data points:

(1) $$\Delta S_{m}=\sum_{i}\frac{M(T+\Delta T)-M(T)}{\Delta T}\Delta H_{i}$$

**Figure 6:**
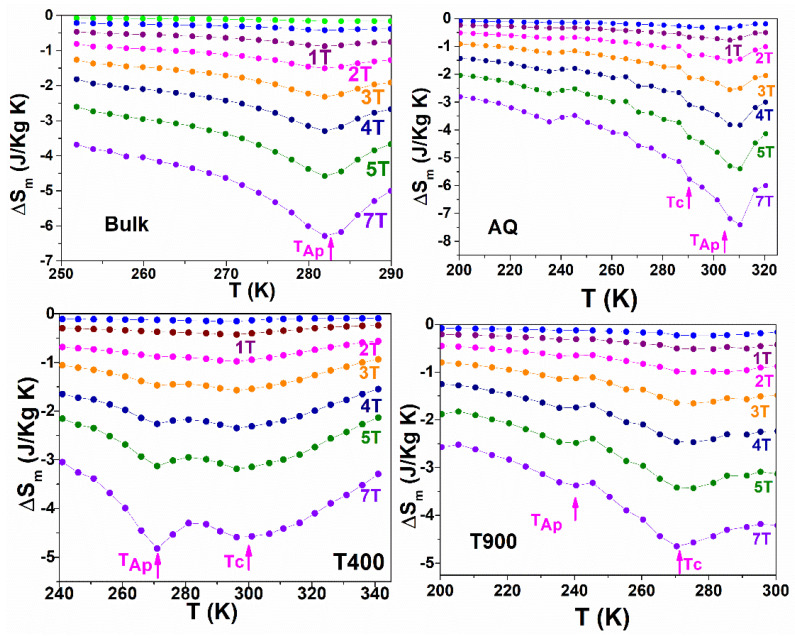
Magnetic entropy variation ΔS_m_ versus raising temperature.

Figure 6 highlights the differences between the magnetic entropy variation in the bulk and the AQ, T400, and T900 ribbons. It follows from relation (1) that ΔS_m_ depends on the rate of variation in magnetization with temperature and this is maximum at the Curie temperature. However, the short-range order that is preserved between the magnetic moments and above the Curie temperature makes the magnetization cancellation experimentally possible over a relatively wide temperature range. That is why the contribution of the Curie temperature to ΔS_m_ takes place over a relatively broad temperature range. Bulk and AQ samples have T_C_ and MT in the same temperature range. Thus, for 5 T applied field, the as-prepared ribbons show ΔS_m_ = −5.41 J/kgK (at 310 K) and bulk alloy −4.58 J/kgK (at 282 K). The variation in the magnetic entropy has a different behavior for the T400 and T900 samples. For these, ΔS_m_ shows a maximum (in absolute value) in the specific MT temperature range and a second peak is clear in the Curie temperature region. In addition, the martensitic transformation is highlighted on Figure 6 by the specific peak temperature of the austenite phase (T_Ap_) obtained on the reverse martensite–austenite transformation by DSC. The Curie temperatures of the studied alloys are also marked with magenta arrows. The values obtained for the variation in the magnetic entropy change in the same trend as that reported in the literature [44,45,46,47].

**Figure 7:**
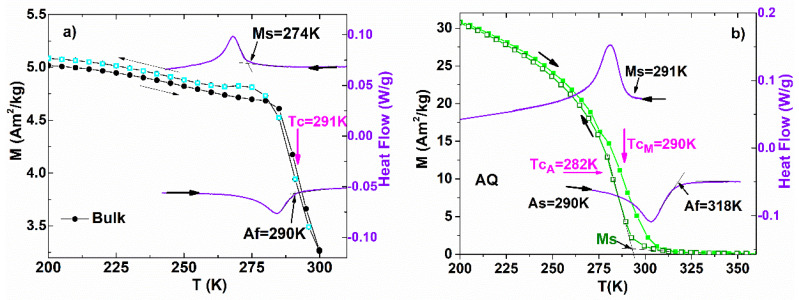
Temperature dependence of magnetization and DSC scans for (**a**) Bulk and (**b**) AQ ribbons.

The results of the DSC analysis and of the thermal variation in the magnetization in 0.2 T applied magnetic field, in the Bulk alloy (Figure 7a) and the same for AQ ribbons (Figure 7b), have been represented in order to identify the thermal overlap of the MT with that of the Curie temperature and their contributions to the ΔS_m_. The Curie temperature of the Bulk alloy (obtained from the first derivative of the magnetization in relation to the temperature dM/dT) is equal to the austenite finish temperature Af = 290 K. Therefore, the alloy is FSMA and shows consecutive phase transformations (the magnetic and structural transitions are successive, in close vicinity). From Figure 7b, it can be seen that the Curie temperature of martensite T_CM_ (determined from dM/dT on the heating curve) is equal to the starting temperature of martensite Ms = 290 K, indicating the ferromagnetic ordering of martensite. At the same time, the Curie temperature of austenite (determined from dM/dT on the cooling curve) T_CA_ = 282 K is lower than the austenite start As = 290 K, yielding a paramagnetic austenite. The AQ ribbons present concomitant magnetic transitions and phase transformation, because the MT occurred between paramagnetic austenite and ferromagnetic martensite. This behavior explains the higher value of the magnetocaloric effect obtained on the AQ bands and is in agreement with the literature [35] which emphasizes the role of magnetostructural transformation.

### 3.6. MR Evaluation from Ac Resistivity (ρ)

Another effect that gives a multifunctional character to ferromagnetic Heusler alloys with shape memory effect is the magnetoresistive effect (MR). This is due to the variation in the electrical resistivity under the effect of the magnetic field in the thermal domain of the martensitic transformation. According to Barandiaran et al. [48], the magnetoresistance anomaly in the range of martensitic transformation is the result of the combination between the change in the resistance during the phase transformation and the change in the transformation temperature under the effect of the magnetic field.

**Figure 8:**
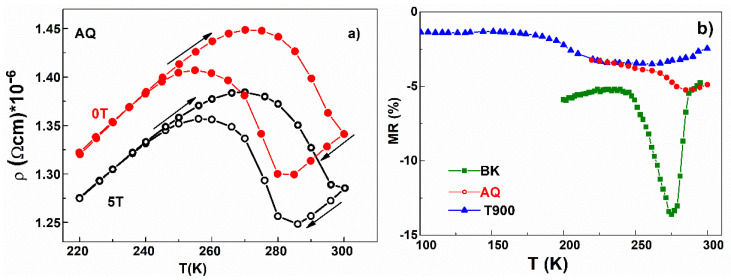
(**a**) Temperature dependence of resistivity in H = 0 T and H = 5 T (open symbols) for AQ ribbons; (**b**) Thermal variation in magnetoresistance in 5 T.

To characterize the magnetoresistive effect, the variation in electrical resistivity was measured as a function of temperature, in the temperature range specific to the martensitic transformation of each sample, without an applied magnetic field (0 T) and, respectively, in a magnetic field of 5 T. In Figure 8a, the temperature dependence of the resistivity for the AQ ribbons in both cases is shown. Temperature dependence of resistivity reveals a metallic behavior in both austenite and martensite, with a jump along the MT transformation, explained by the increase in the scattering on the boundaries of the martensite-specific of twin-variants. In addition, it is observed that the resistivity decreases when the magnetic field is applied [47,48,49], reflecting the changes made to the resistivity by the reduction in the magnetic disorder when the magnetic field is applied.

Magnetoresistance was calculated using the formula:

(2) $$MR=\frac{\rho (H)-\rho (0)}{\rho (0)},$$

*ρ*(0) and *ρ*(*H*) represent the resistivity values without applied magnetic field and, respectively, for 5 T. The continuous variation in MR% with temperature, corresponding to bulk, AQ and T900 samples, are shown in Figure 8b. The value of the MR obtained for the bulk sample of −13% at 275 K is impressive and is much higher than the values obtained in ribbons, namely −5.23% at 285 K and −3.42% at 235 K for the AQ ribbons and T900 sample, respectively.

## 4. Conclusions

In this study, the Heusler non-stoichiometric Ni_55_Nd_2_Fe_18_Ga_25_ alloy, prepared by the electric arc melting and melt spinning method, was investigated. The effects of the thermal treatments on the phase composition, morphology, and magneto-caloric and magneto-transport properties of the nanostructured ribbons have been examined and compared with the bulk samples. The thermal treatments produce, besides atomic ordering, the segregation of the gamma phase, which depletes the austenitic matrix in 3D elements, hence influencing the thermal positioning of the TM and of the magnetic ordering. Specifically, the Curie temperatures increase for the ordered structure, while the TM characteristic temperatures decrease with the rising TT temperature.

The bulk samples show consecutive phase transformations with the magnetic transition at a higher temperature than the structural transition. Interestingly, however, the as-quenched ribbons show a concomitant magneto–structural transition in the RT range. As a consequence, the AQ ribbons have the highest value of the magnetic entropy variation from all measured samples, of −5.41 J/kgK at 310 K. For the thermally treated ribbons, the magnetic and structural transitions are again separated, and the two maxima occur in the thermal dependence of the magnetic entropy variation in connection with the martensitic transformation and in Curie temperatures.

A remarkable magnetoresistive effect of 13.5% at 275 K was recorded in the bulk sample, which is much higher than in the ribbons (5.23% at 285 K and 3.42% at 235 K for the AQ ribbons and T900 sample, respectively).

## Figures and Tables

**Table 1:** DSC transformation temperatures (Ms, Mf, As, Af), thermodynamic equilibrium temperature T_0_, the Curie temperatures (T_CM_, T_CA_) and magnetic entropy change ΔS_m_ (at 5 T) for studied samples. T_CA_ is determined on cooling and T_CM_ is determined on heating.

Sample	Ms (K)	Mf (K)	As (K)	Af (K)	T_0_ (K)	T_CM_ (K)	T_CA_ (K)	ΔS_m_ (J/kgK)
Bulk	274	262.6	275.6	290	282	300	291	−4.58
AQ	291	269	290	318	304.5	290	282.6	−5.4
T400	267	246	261	285	276	296	291.6	−3.2
T900	258	204	210	281	269.5	275	271	−3.4

**Table 2:** Crystalline phases and lattices parameters, extracted from XRD data.

Sample	Phase	a (Å)	c (Å)	V (Å^3^)	Dp (nm)
Bulk	Fm-3m	5.747 (1)	-	189.8	-
	Fm-3m	3.618 (9)	-	47.4	-
	R-3m	8.565 (1)	12.476 (1)	792.6	-
AQ	P 4/mmm	2.481 (1)	3.515 (9)	21.6	43.3
	Fm-3m	3.549 (9)	-	44.7	21.4
	R-3m	8.557 (4)	12.425 (9)	788	125.1
T400	Fm-3m	5.729 (2)	-	188.1	84.6
	Fm-3m	3.590 (6)	-	46.3	139.9
	R-3m	8.563 (1)	12.449 (9)	790.6	147.5
T900	Fm-3m	5.744 (9)	-	189.6	121.7
	Fm-3m	3.590 (2)	-	46.3	82.2
	R-3m	8.560 (1)	12.459 (2)	790.6	131.3

## Data Availability

Data is available, upon reasonable request, by e-mail addressed to the corresponding author.
